# Supplementary material for: Development of Intracorporeal Differentiation of Stem Cells to Induce One-Step Mastoid Bone Reconstruction during Otitis Media Surgeries
Source: Polymers (Basel). 2022 Feb 23;14(5):877. doi: 10.3390/polym14050877 (PMC8912861; doi:10.3390/polym14050877)
Supplement: Supplementary file 1 [file polymers-14-00877-s001.zip › polymers-1578589-supplementary.pdf]

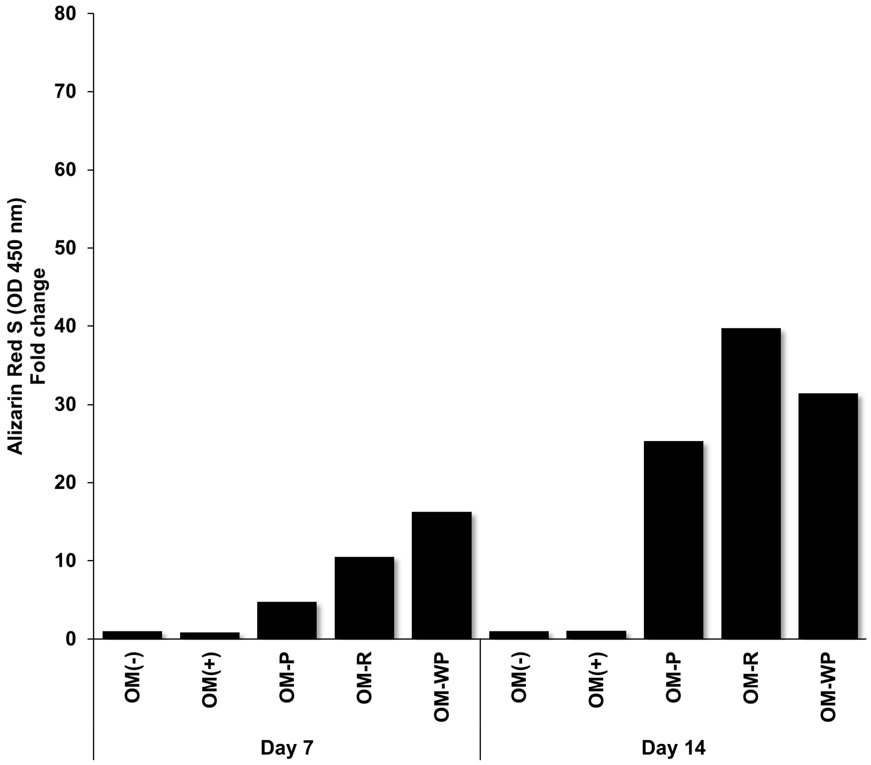

Figure S1. The quantitative results of retention of Alizarin Red S corresponding the previous phenotypic data. Osteogenic media (OM) (-): Low glucose (1g/L) DMEM, 1% penicillin/streptomycin with Dexamethasone (0.1 μM final), L-Ascorbic acid 2-phosphate (50.0 μM final) and β-glycerophosphate (10.0 mM final). OM (+): OM with 10 % FBS, OM-P: OM with 10 % Platelet poor plasma, OM-R: OM with 10 % Platelet rich plasma, OM-WP: OM with 10 % Whole plasma.
